# The impact of 27-hydroxycholesterol on endometrial cancer proliferation

**DOI:** 10.1530/ERC-17-0449

**Published:** 2018-01-25

**Authors:** Douglas A Gibson, Frances Collins, Fiona L Cousins, Arantza Esnal Zufiaurre, Philippa T K Saunders

**Affiliations:** Medical Research Council Centre for Inflammation ResearchThe University of Edinburgh, Queen’s Medical Research Institute, Edinburgh, UK

**Keywords:** LXR, ER, 27-hydroxycholesterol, proliferation, endometrial cancer

## Abstract

Endometrial cancer (EC) is the most common gynaecological malignancy. Obesity is a major risk factor for EC and is associated with elevated cholesterol. 27-hydroxycholesterol (27HC) is a cholesterol metabolite that functions as an endogenous agonist for Liver X receptor (LXR) and a selective oestrogen receptor modulator (SERM). Exposure to oestrogenic ligands increases risk of developing EC; however, the impact of 27HC on EC is unknown. Samples of stage 1 EC (*n* = 126) were collected from postmenopausal women undergoing hysterectomy. Expression of LXRs (*NR1H3*, LXRα; *NR1H2*, LXRβ) and enzymes required for the synthesis (*CYP27A1*) or breakdown (*CYP7B1*) of 27HC were detected in all grades of EC. Cell lines originating from well-, moderate- and poorly-differentiated ECs (Ishikawa, RL95, MFE 280 respectively) were used to assess the impact of 27HC or the LXR agonist GW3965 on proliferation or expression of a luciferase reporter gene under the control of LXR- or ER-dependent promoters (LXRE, ERE). Incubation with 27HC or GW3965 increased transcription via LXRE in Ishikawa, RL95 and MFE 280 cells (*P* < 0.01). 27HC selectively activated ER-dependent transcription (*P* < 0.001) in Ishikawa cells and promoted proliferation of both Ishikawa and RL95 cells (*P* < 0.001). In MFE 280 cells, 27HC did not alter proliferation but selective targeting of LXR with GW3965 significantly reduced cell proliferation (*P* < 0.0001). These novel results suggest that 27HC can contribute to risk of EC by promoting proliferation of endometrial cancer epithelial cells and highlight LXR as a potential therapeutic target in the treatment of advanced disease.

## Introduction

Endometrial cancer (EC) is the most common gynaecological malignancy and the fourth most common cancer in women in developed countries with incidence increasing in line with rising rates of obesity (reviewed in Onstad* et al*. 2016). Obesity is a major modifiable risk factor for EC and is thought to contribute to increased risk of malignancy in part due to increased exposure to estrogens, which enhance the risk of aberrant proliferation within the endometrium (Sanderson* et al*. 2017). Obesity is also associated with an adverse metabolic profile, which is postulated to independently increase risk of EC (Trabert* et al*. 2015).

A recent meta-analysis supported a positive association between dietary cholesterol consumption and endometrial cancer risk (Gong* et al*. 2016). Notably, obesity also puts individuals at risk of developing an adverse, raised, cholesterol profile. Cholesterol metabolites such as the oxysterol 27-hydroxycholesterol (27HC) have been demonstrated to promote cancer growth and metastasis in studies on breast cancer (Nelson* et al*. 2013, Wu *et al*. 2013), providing a plausible mechanistic link between increased adiposity and EC risk. 27HC is a primary metabolite of cholesterol, synthesised by the action of sterol 27-hydroxylase (CYP27A1) and metabolised by 25-hydroxycholesterol 7-α-hydroxylase (CYP7B1; (1)). 27HC acts as an endogenous agonist for the Liver X receptor (LXR), a ligand-activated transcription factor involved in the regulation of cholesterol homeostasis. Two isoforms of LXR have been identified; LXRα (encoded by *NR1H3*), which is predominantly expressed in the liver, kidney and small intestine but exhibits low expression in other tissues, and LXRβ (encoded by *NR1H2*), which is ubiquitously expressed. Based largely on studies in breast cancer, LXRs have been proposed as a novel anti-cancer target and the LXR-selective agonists GW3965 and bib901317 are reported to decrease proliferation of LXR-expressing breast cancer cell lines (MCF7, T47D, MDA-MB231) as well as the prostate cancer cell line LNCaP (Vedin* et al*. 2009, Kim* et al*. 2010). To the best of our knowledge, LXR expression has not been reported in human EC tissues and the impact of either 27HC or LXR agonists on the endometrium or endometrial malignancies is not known.

In addition to activating LXRs, 27HC can also bind oestrogen receptors (ER) (Umetani* et al*. 2007) and acts as an endogenous selective oestrogen receptor modulator (SERM) (DuSell* et al*. 2008). 27HC has diverse impacts and its SERM activity is reported to be both tissue selective and context dependent. For example, 27HC acts as a competitive antagonist of ERs expressed in the vasculature and can antagonise E2-mediated endothelial cell migration and re-endothelialisation (Umetani *et al*. 2007). In contrast, in the absence of E2, 27HC is reported to act as an agonist to ERα (ESR1) to increase cell adhesion and expression of pro-inflammatory cytokines such as tumour necrosis factor alpha (TNFA) and interleukin 6 (IL6) (Umetani* et al*. 2014) by endothelial cells and macrophages. Notably, 27HC is also reported to increase proliferation of ERα-positive breast cancer cell lines and promotes MCF7 tumour xenograft growth in mice by stimulating ER-dependent cell proliferation (Wu *et al*. 2013). Given selective LXR agonists have anti-proliferative effects (Vedin *et al*. 2009), these studies suggest that proliferative effects of 27HC may be mediated via ER and that relative expression of LXR or ER isoforms may define the impact of the ligand.

ER isoforms are expressed in EC tissues and ER expression changes with disease progression (Collins* et al*. 2009). We have previously reported that ERα is readily detectable in both epithelial and stromal cells in well-differentiated cancers but is significantly reduced in poorly differentiated cancers. In contrast, expression of ESR2 variants (ERβ1, 2, 5) was readily detected in well, moderate and poorly differentiated stage 1 ECs (Collins *et al*. 2009). We therefore postulated that 27HC might have distinct effects in EC depending on the bioavailability of ER isoforms present at different stages of disease progression.

Obesity and the metabolic syndrome are both associated with an increased risk of developing ­pre-malignant and malignant endometrial disease (Sanderson *et al*. 2017) but whether the cholesterol metabolite 27HC has an impact on EC risk/progression is not known. In the current study, we assessed the expression of the enzymes required for synthesis (CYP27A1) and breakdown (CYP7B1) of 27HC and assessed expression of the cognate receptors LXRα and LXRβ in primary human stage I endometrial adenocarcinomas (*n* = 126) and postmenopausal endometrial controls (*n* = 9). The impact of 27HC and the LXR-selective agonist GW3965 on ERE- and LXRE-dependent expression of a reporter gene, as well as cellular proliferation, was assessed in three EC cell lines which phenocopy well-, moderate- and poorly differentiated stage I ECs. Our novel findings demonstrate that 27HC can alter responses in EC cells and highlight LXR as a potential therapeutic target. Taken together, our findings suggest increased exposure to 27HC may increase risk of development and progression of EC.

## Materials and methods

### Human tissue samples

Endometrial adenocarcinoma tissue was collected from postmenopausal women undergoing total abdominal hysterectomy who had been previously diagnosed to have endometrioid adenocarcinoma of the endometrium; they had received no treatment before surgery (Supplementary Table 1, see section on supplementary data given at the end of this article). Written informed consent was obtained from all subjects prior to surgery, and ethical approval was granted by the Lothian Research Ethics Committee (LREC1999/6/4). Methods were carried out in accordance with NHS Lothian Tissue Governance guidelines. All ECs were confined to the uterus (International Federation of Obstetrics and Gynaecology, FIGO, stage 1 as described in Collins *et al*. 2009). Diagnosis of adenocarcinoma was confirmed histologically by an experienced gynaecological pathologist, and tissues were further graded as well differentiated (G1), moderately differentiated (G2) or poorly differentiated (G3). Samples were anonymised and patient follow-up information was not available. However, survival statistics for stage 1 EC in the UK are reported as 99% 1-year survival and 95.3% 5-year survival (Cancer Research UK; http://www.cancerresearchuk.org/health-professional/cancer-statistics/statistics-by-cancer-type/uterine-cancer – accessed November 2017) and Information Services Division Scotland figures, which cover the stage 1 EC samples collected in the current study, report 92.9% 1-year survival and 83.2% 5-year survival for all uterine cancers (http://www.isdscotland.org/Health-Topics/Cancer/Cancer-Statistics/Female-Genital-Organ/#uterus – accessed November 2017).

Postmenopausal controls (*n* = 9) were obtained from women undergoing surgery for non-malignant gynaecological conditions. None of the women were receiving hormonal therapy. A total of 126 EC tissue samples were analysed; 3 samples per grade were assessed for immunohistochemistry and *n* = 30 well-differentiated cancers, *n* = 64 moderately differentiated and *n* = 32 poorly differentiated samples were assessed for qPCR studies. A minimum of 10 samples at each grade were analysed for each gene, detailed sample numbers are included in Supplementary Table 2. Tissue for immunohistochemistry was collected in neutral buffered formalin (NBF), RNA extraction samples were collected in RNALater (Qiagen).

### Measurement of mRNA

Isolation of mRNAs, preparation of cDNAs and analysis by qPCR were performed according to standard protocols (Bombail* et al*. 2010); samples were quantified by relative standard curve method or by the comparative ΔΔCt method with *CYC* as internal control. Primers/probes are given in Supplementary Table 3.

### Immunohistochemistry

Single antibody immunohistochemistry using 3,3′-diaminobenzidine tetra-hydrochloride (DAB) detection was performed as described previously (Collins *et al*. 2009). Double immunofluorescence was carried out with antibodies directed against LXR or ERα and the proliferation marker Ki67. Details of antibodies and dilutions are provided in Supplementary Table 4. Primary antibodies were incubated at 4°C overnight. Antigen detection was performed using Tyramide signal amplification (Perkin Elmer) system followed by nuclear counterstaining with DAPI (4′,6-diamidino-2-phenyl-indole dihydrochloride). Negative controls were incubated in the absence of primary antibody but otherwise processed as above; no staining was detected in no primary controls for any of the antibodies used (not shown). Images were captured using a LSM 710 Confocal microscope (Zeiss) at ×40 magnification.

### Cell cultures

Three endometrial adenocarcinoma cell lines representative of well-, moderately- or poorly differentiated cancers were used. Ishikawa cells were obtained from the European Collection of Cell Culture (ECACC no. 99040201, Wiltshire, UK). This cell line was originally derived from a well-differentiated adenocarcinoma of a 39-year-old woman (Nishida* et al*. 1985) and reported to express both ERα and ERβ protein (Johnson* et al*. 2007). RL95-2 cells (ATCC CRL-1671; hereafter RL95) were originally derived from a Grade 2 moderately differentiated endometrial adenocarcinoma (Way* et al*. 1983) and reported to express both ERα and ERβ protein (Yang* et al*. 2008, Li* et al*. 2014). MFE-280 (ECACC no. 98050131) were derived from a recurrent, poorly differentiated, endometrial adenocarcinoma and have low/undetectable expression of ERα and ERβ. Cells were maintained in DMEM/F12 (Sigma) supplemented with 10% FBS, 100 U penicillin, streptomycin and 0.25 µg/mL fungizone (Invitrogen) at 37°C in 5% CO_2_. Media for RL95 was supplemented with 0.005 mg/mL insulin (Sigma). Cells were incubated with 27-hydroxycholesterol (27HC; Tocris Cat. No. 3907) using stocks diluted in ethanol to give final concentrations ranging from 10^−5^ M to 10^−8^ M or GW 3965 hydrochloride (GW; Tocris Cat. No. 2474) using stocks diluted in DMSO to give final concentrations ranging from 10^−5^ M to 10^−8^ M. Some cultures were co-incubated with the anti-estrogen fulvestrant (ICI 182,780; Tocris Cat. No. 1047) diluted in DMSO at a final concentration of 10^−6^ M. Appropriate vehicle control incubations were included in all studies. All cell lines were authenticated using the Promega PowerPlex 21 system (Eurofins Genomics, Ebersberg, Germany).

### Reporter assays

An adenoviral vector containing a 3× ERE-tk-luciferase reporter gene was prepared as described previously (Collins *et al*. 2009). Cells were cultured in DMEM without phenol red and containing charcoal stripped foetal calf serum (CSFCS) for 24 h before being infected with Ad-ERE-Luc at a MOI of 25. Activation of LXR-dependent signal transduction was assessed according to manufacturer’s instructions using reagents from the Cignal LXR Reporter Kit, which includes positive and negative controls as well as a luciferase reporter gene under the control of tandem repeats of the LXR transcriptional response element (LXRE) (Qiagen, CCS-0041L).

Cells were treated for 24 h and luciferase activities were determined using ‘Bright Glo’ reagents (Promega). Luminescence was measured using Fluostar Microplate Reader (BMG labtech) and fold-change in luciferase activity was calculated relative to vehicle control for each treatment.

### Proliferation assays

The impact of treatments on cell proliferation was assessed using CyQUANT Direct Cell Proliferation Assay (Thermo Fisher, C35011) according to manufacturer’s instructions and nuclear fluorescence measured using Novostar Microplate Reader (BMG labtech). For each cell line investigated, cell number was quantified using a standard curve of known cell numbers and fold-change in cell number calculated relative to vehicle control for each treatment.

### Statistical analysis

Statistical analysis was performed using GraphPad prism. One-way ANOVA was used to determine significance between treatments in data that were normally distributed. Non-parametric testing was utilised where sample sizes were insufficient to confirm normality of data distribution; Kruskal–Wallis test was used to assess differences between treatments. Where data were analysed as fold-change, significance was tested using one sample *t* test and a theoretical mean of 1. Criterion for significance was *P* < 0.05. All data are presented as mean ± s.e.m.

## Results

### Enzymes that regulate bioavailability of 27-hydroxycholesterol and its cognate receptor LXR are expressed in EC

**Figure 1:**
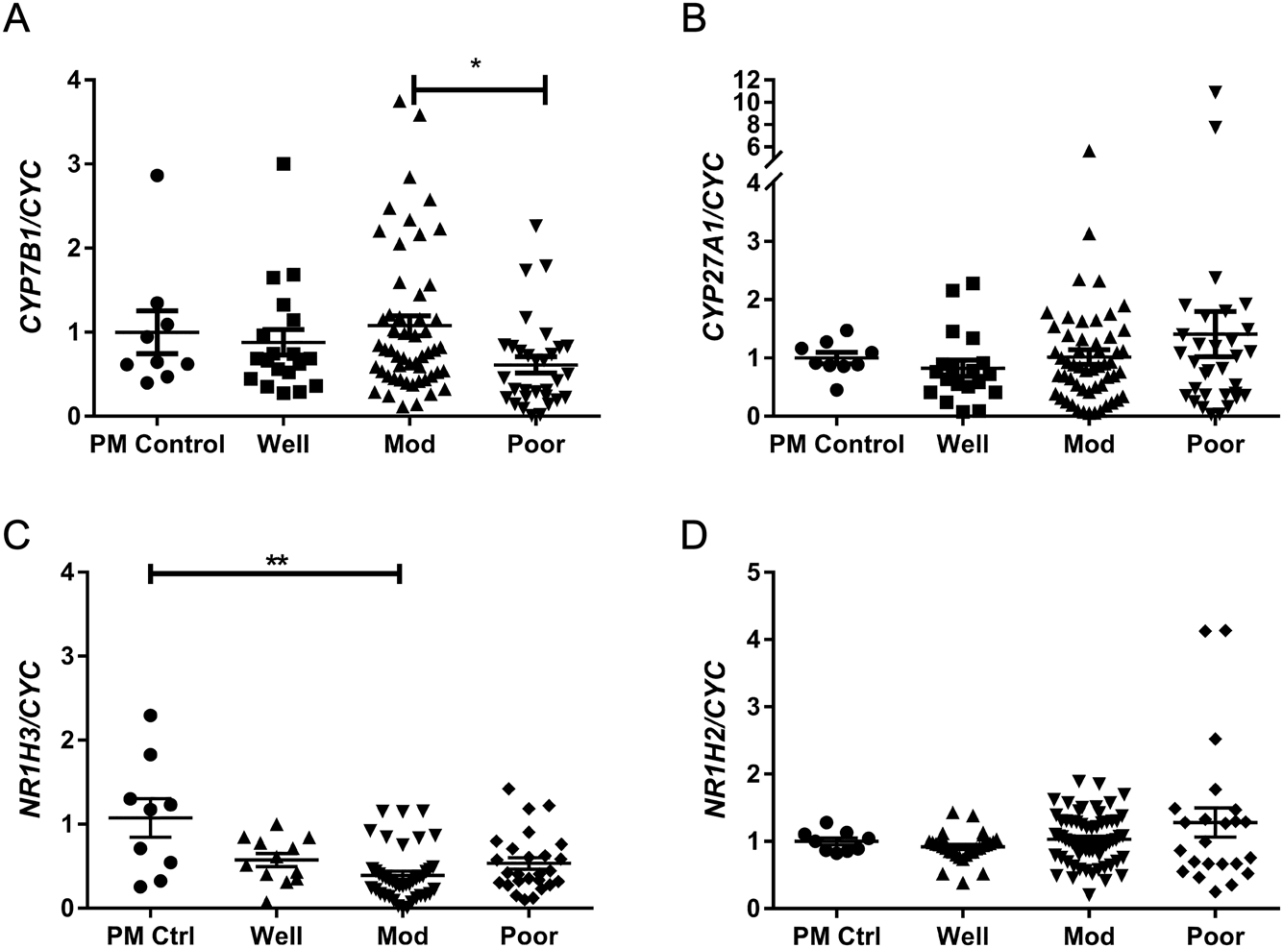
27HC signalling pathway is expressed in endometrial cancer and altered with disease severity. The expression of *CYP7B1*, *CYP27A1*, *NR1H3* (LXRα) and *NR1H2* (LXRβ) relative to internal control gene *CYC* was assessed by qPCR in postmenopausal control endometrium (PM Ctrl) and in endometrial cancer tissue homogenates from well-, moderately- and poorly differentiated endometrial adenocarcinomas. Relative expression of mRNAs encoding *CYP7B1* (A) were decreased in poorly differentiated cancers compared to moderately differentiated cancers but *CYP27A1* was not significantly different (B). Relative expression of mRNAs encoding *NR1H3* (C; LXRα) were significantly decreased in moderately differentiated cancers compared to postmenopausal control tissues whilst *NR1H2* (LXRβ) was not significantly different (D). *P < 0.05, **P < 0.01. Kruskal–Wallis test with multiple comparisons. PM, *n* = 9; Well *n* = 12–30; Mod, *n* = 42–64; Poor, *n* = 23–32. All data are presented as mean ± s.e.m.

Messenger RNAs encoded by *CYP7B1* and *CYP27A1* were detected in all cancer grades (Fig. 1A and B); expression of *CYP7B1* was significantly lower in poorly differentiated cancers compared to moderately differentiated cancers (*P* < 0.05). Relative expression of *CYP27A1* tended to be higher in poorly differentiated cancers, but this was not significant. We next assessed relative expression of mRNAs encoding the LXR receptors known to bind 27HC: *NR1H3* (LXRα) and *NR1H2* (LXRβ) were detected in all cancer grades (Fig. 1C and D). Expression of *NR1H3* was significantly lower in moderately differentiated cancers compared to postmenopausal controls (*P* < 0.01). Expression of *NR1H2* did not change between sample groups.

### Immunolocalisation of LXR and the proliferation marker Ki67 in EC tissue sections

**Figure 2:**
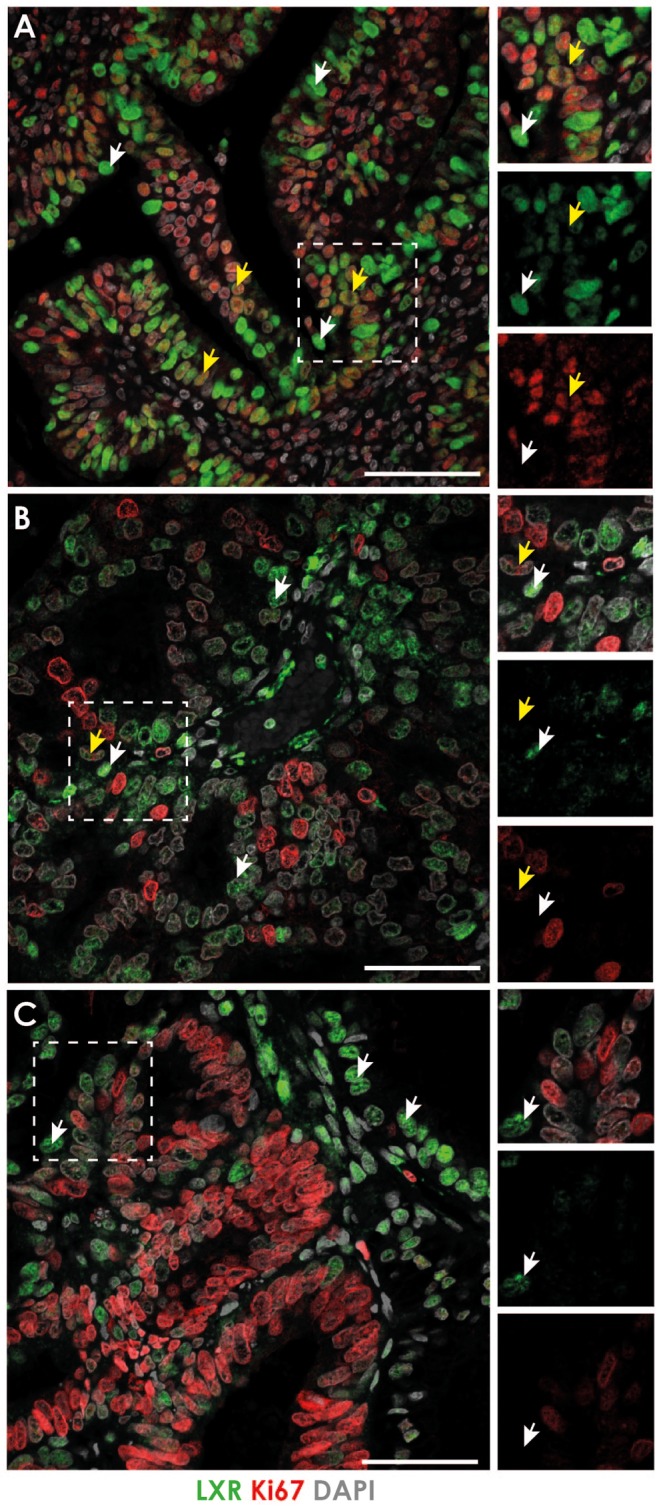
Expression of LXR and the proliferation marker Ki67 in endometrial cancer. The expression of LXR (antibody identified both isoforms) and the proliferation marker Ki67 was assessed by immunohistochemistry in endometrial cancer tissue sections. In well-differentiated cancers (A), LXR was expressed throughout the tissue and localised to the nuclei of both stromal and epithelial cells (green staining). Nuclear immunoexpression of Ki67 (red staining) was detected and co-localised with LXR expression (yellow arrows) although some LXR-positive cells did not co-express Ki67 (white arrows). In moderately differentiated cancers (B) both markers were detected but did not appear to co-localise; only few cells expressed both LXR and Ki67 (yellow arrows). Most LXR-positive cells did not co-express Ki67 (white arrows). This was also true of poorly differentiated cancers (C), few cells expressed both LXR and Ki67 (yellow arrows) although LXR-positive cells were found in close association with proliferating cells (white arrows). Images representative of at least 3 different patients per cancer grade. Nuclear counterstain DAPI (grey). All scale bars 50 µM.

The expression of LXR in EC tissue sections was assessed by immunohistochemistry using an antibody that detected both isoforms of LXR (mouse anti-LXR; sc-271064). LXR was readily detected in well-, moderately- or poorly differentiated cancers and was immunolocalised to both stromal and epithelial cells (Supplementary Fig. 1). To assess if LXR expression was associated with cell proliferation within EC tissue, we performed double immunofluorescence staining for both LXR and the proliferation marker Ki67 (Fig. 2). In well-differentiated cancers (Fig. 2A), nuclear immunoexpression of Ki67 (red staining) was detected which co-localised (yellow arrows) with LXR expression (green staining, note that single channel views show that the intensity of LXR staining varied between cells). Whilst careful evaluation of single channel views confirmed that the majority of LXR-positive cells were also immunopositive for Ki67 some cells were Ki67 negative (white arrows). In contrast, in moderately differentiated cancers (Fig. 2B), both markers were detected but few cells appeared to co-localise (yellow arrows) although LXR-positive cells (white arrows) were found in close association with proliferating cells. In poorly differentiated cancers (Fig. 2C), few cells expressed both markers. Ki67-positive cells were clustered in regions with limited LXR expression and no co-expression of LXR and Ki67 was detected. LXR^+^Ki67^−^ cells (white arrows) were detected close to Ki67^+^ cells. We also assessed the expression of ERα and Ki67 in EC tissues (Supplementary Fig. 2) as this receptor is implicated in the regulation of proliferation in normal endometrium (Lubahn* et al*. 1993, Frasor* et al*. 2003). Consistent with our previous study, ERα was not detected in the poorly differentiated cancers (Collins *et al*. 2009) and immunoexpression of Ki67 was clearly independent of ERα with an increase in abundance of positive nuclei in poor (sample codes 910/2178) as compared to well or moderately differentiated tissue where co-localisation of ERα and Ki67 was readily detected.

### 27HC activates LXRE- and ERE-dependent transcription in endometrial epithelial cancer cells and alters proliferation of EC cells

**Figure 3:**
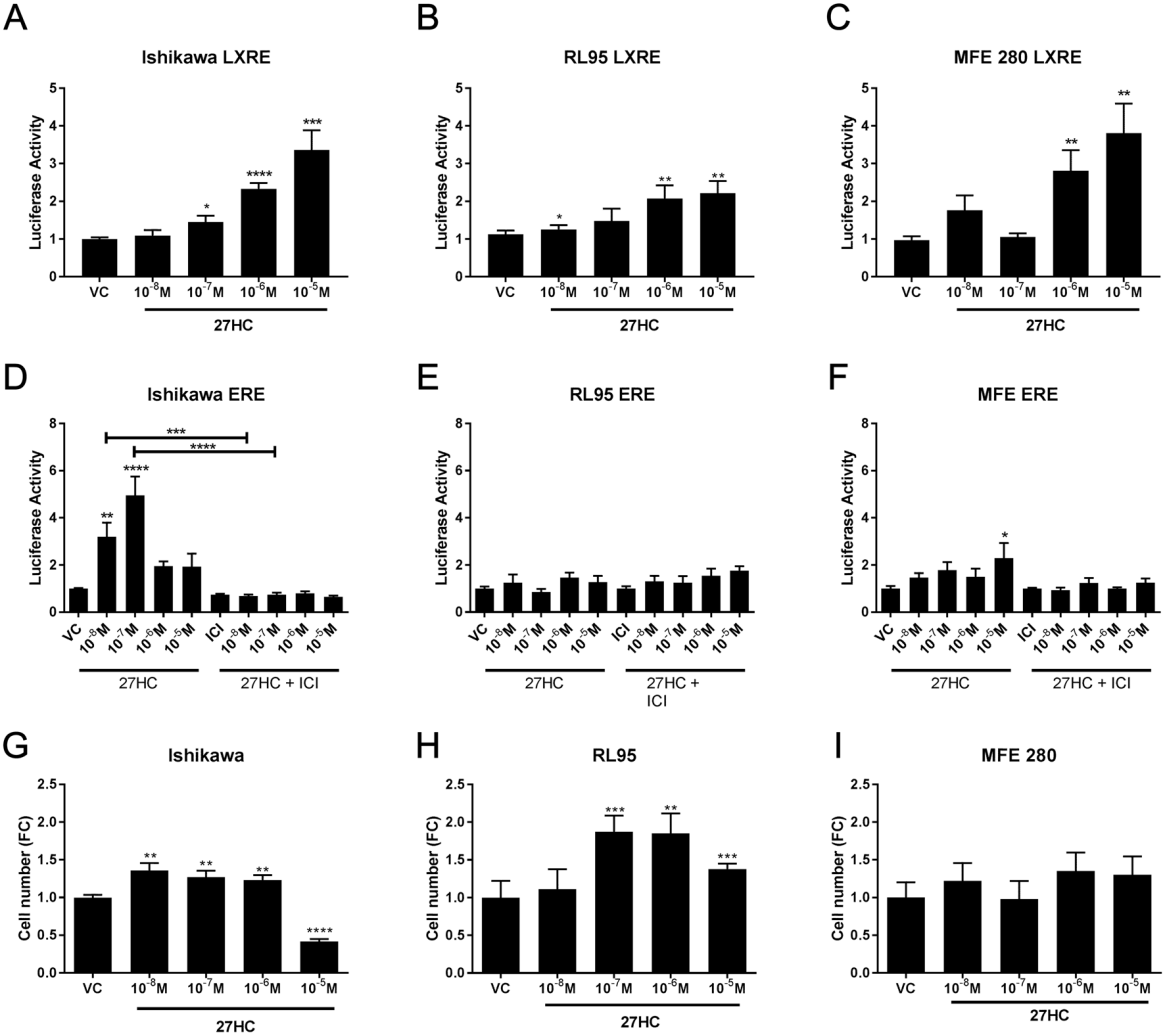
27HC activates LXRE- and ERE-dependent transcription in endometrial epithelial cancer cells and alters proliferation. The cholesterol metabolite 27-hydoxycholesterol (27HC) is the endogenous agonist for LXR and is also classified as selective oestrogen receptor modulator. The impact of 27HC on LXRE- (A, B and C) and ERE-dependent (D, E and F) transcription was investigated by luciferase reporter assay in endometrial cancer cell lines; Ishikawa, RL95 and MFE280. 27HC significantly increased LXRE-dependent transcription in a dose-dependent manner in each endometrial cancer cell line. 27HC stimulated ERE-dependent transcription only at lower concentrations and was significantly increased by 10^−8^ M 27HC (*P* < 0.01) and maximally stimulated by 10^−7^ M 27HC (*P* < 0.0001). The 27HC effect was abrogated by co-incubation with the antiestrgoen Fulvestrant (ICI 182,780; ICI) at all concentrations of 27HC (D). 27HC did not increase ERE-dependent transcription in RL95 (E) and was only increased by 10^−5^ M 27HC (*P* < 0.05) in MFE280 cells (F). Cell proliferation was assessed by CyQuant direct proliferation assay in each cell line (G, H and I). Proliferation of Ishikawa cells was increased by 10^−8^ M (*P* < 0.01), 10^−7^ M (*P* < 0.01) and 10^−6^ M (*P* < 0.01) 27HC but decreased by 10^−5^ M 27HC (*P* < 0.0001; G). Proliferation of RL95 cells was increased by 10^−7^ M (*P* < 0.001), 10^−6^ M (*P* < 0.01) and 10^−5^ M (*P* < 0.001) 27HC (H). 27HC did not affect proliferation in MFE280 cells (I). *P < 0.05, **P < 0.01, ***P < 0.001, ****P < 0.0001. One sample *t* test and a theoretical mean of 1. All data are presented as mean ± s.e.m.

Having demonstrated expression of enzymes and receptors required for 27HC signalling, we extended our observational study by exploring the impact of the ligand on endometrial epithelial cancer cell lines chosen to model well-, moderately- or poorly differentiated stage I cancers; Ishikawa, RL95 and MFE 280. Protein expression of both LXR isoforms was confirmed by western blot in all cell lines studied (Supplementary Fig. 3A and B). We assessed the mRNA expression of LXRs in these cell lines and found that their expression phenocopied that found in tissue samples (Supplementary Fig. 3). *NR1H3* mRNA expression was significantly decreased in RL95 (moderately differentiated) cells compared to MFE 280 (poorly differentiated; *P* < 0.01; Supplementary Fig. 3C). Consistent with tissue mRNA expression patterns, *NR1H2* was not different between cell lines (Supplementary Fig. 3D). Messenger RNAs encoded by both ER genes; ERα (*ESR1*) and ERβ (*ESR2*; ERβ1 specific primers) were detected in all of the cell lines (Supplementary Fig. 4). *ESR1* mRNAs were significantly reduced in RL95 and MFE280 compared to Ishikawa cells (Supplementary Fig. 4A) consistent with patterns of expression in intact tissue (Supplementary Fig. 2). *ESR2* mRNA was significantly reduced in MFE280 cells compared to Ishikawa (Supplementary Fig. 4B). As 27HC is both an endogenous agonist for LXR and a SERM, the impact of 27HC on LXRE- and ERE-dependent transcription was investigated in the EC cell lines. 27HC significantly increased LXRE-dependent transcription in a dose-dependent manner in all 3 cell lines and was maximally stimulated by 10^−5^ M 27HC (Fig. 3A, B and C). In contrast, 27HC only stimulated ERE-dependent transcription in Ishikawa cells (Fig. 3D) at 10^−8^ M (*P* < 0.01) and 10^−7^ M (*P* < 0.0001). The impact of 27HC was abrogated by co-incubation with the anti-oestrogen fulvestrant (ICI 182,780) consistent with ER dependence. In contrast to Ishikawa cells, 27HC had little impact on ERE-dependent transcription in RL95 (Fig. 3E) and MFE280 cells (Fig. 3F). As 27HC could activate both ERE- and LXRE-promoters, we assessed its impact on cell proliferation (Fig. 3G, H and I). 27HC induced proliferation of Ishikawa cells at concentrations ranging from 10^−8^ M to 10^−6^ M (*P* < 0.01), but this was inhibited at the highest concentration (10^−5^ M, *P* < 0.0001). 27HC significantly increased proliferation in RL95 cells at concentrations of 10^−7^ M (*P* < 0.001) or greater. In contrast, 27HC did not alter proliferation of MFE 280 cells at any of the concentrations investigated. Neither RL95 nor MFE280 cell lines expressed *CYP7B1* (Supplementary Fig. 3E and F) precluding the potential for *in vitro* metabolism limiting cell responses to 27HC in these cell lines.

### Targeting LXR with the synthetic agonist GW3965 activates LXRE-dependent transcription and alters cell proliferation in a cell-specific manner

**Figure 4:**
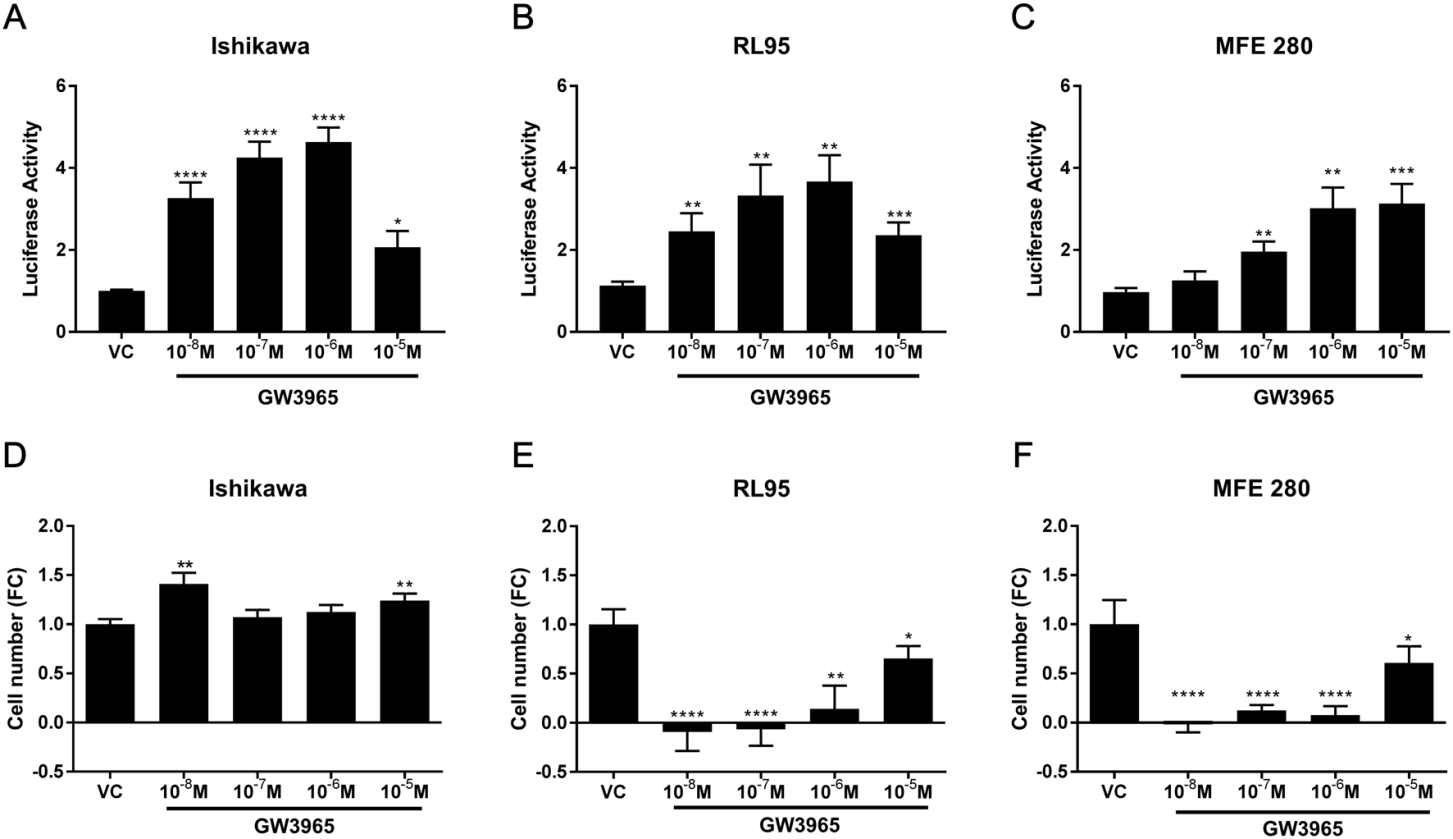
LXR agonist GW3965 activates LXRE-dependent transcription and alters proliferation in endometrial epithelial cancer cells. The impact of the LXR synthetic agonist GW3965 on LXRE-dependent transcription (A, B and C) and on cell proliferation (D, E and F) was assessed in endometrial cancer cell lines; Ishikawa, RL95 and MFE280. GW3965 significantly increased LXRE-dependent transcription in a dose-dependent manner in each endometrial cancer cell line. GW3965 significantly increased LXRE-dependent transcription at all concentrations assessed and was maximally increased by 10^−6^ M GW3965 in Ishikawa cells (*P* < 0.0001) (A) and RL95 cells (*P* < 0.01) (B). LXRE-dependent transcription and was not increased by 10^−8^ M GW3965 but maximally increased by 10^−5^ M 27HC (*P* < 0.001) in MFE280 cells (C). Cell proliferation was assessed by CyQuant direct proliferation assay in each cell line (D, E and F). Proliferation of Ishikawa cells was increased by 10^−8^ M (*P* < 0.01) and by 10^−5^ M 27HC (*P* < 0.01) (D). In contrast, proliferation of RL95 cells was decreased by 10^−8^ M (*P* < 0.001), 10^−7^ M (*P* < 0.001), 10^−6^ M (*P* < 0.01) and 10^−5^ M (*P* < 0.05) GW3965 (E). Proliferation of MFE280 cells was decreased by 10^−8^ M (*P* < 0.001), 10^−7^ M (*P* < 0.0001), 10^−6^ M (*P* < 0.0001) and 10^−5^ M (*P* < 0.05) GW3965 (I). *P < 0.05, **P < 0.01, ***P < 0.001, ****P < 0.0001. One sample *t* test and a theoretical mean of 1. All data are presented as mean ± s.e.m.

Incubation of cells with the LXR-selective agonist GW3965 significantly increased LXRE-dependent transcription in a dose-dependent manner (Fig. 4) consistent with expression of LXRs in the EC cell lines (Supplementary Fig. 3). In contrast to 27HC, GW3965 significantly and robustly increased LXRE-dependent transcription at concentrations ≥10^−8^ M in Ishikawa (Fig. 4A) and RL95 (Fig. 4B) and ≥10^−7^ M in MFE280 cells (Fig. 4C). Although LXR reporter responses were similar in the different cell lines, proliferation responses were strikingly different. In Ishikawa cells, treatment with GW3965 at concentrations 10^−8^ M (*P* < 0.01) and 10^−5^ M (*P* < 0.01) significantly increased proliferation (Fig. 4D). In contrast, GW3965 significantly and robustly decreased cell proliferation at all concentrations investigated in both RL95 (Fig. 4E) and MFE 280 cells (Fig. 4F).

## Discussion

To date, no study has assessed the association between the cholesterol metabolite 27HC and EC. EC incidence rates have increased by ~50% since the early 1990s and approximately 57% of endometrial cancers in the United States have been attributed to being overweight or obese (Cancer Research, UK; http://www.cancerresearchuk.org – accessed November 2017, and Calle & Kaaks 2004). Although increased exposure to adipose-derived estrogens is believed to increase aberrant proliferation within the endometrium (Zhao* et al*. 2016), recent evidence supports an independent role for obesity-associated metabolic factors in modulating EC risk. Notably, both elevated triglycerides and increased dietary cholesterol consumption are reported to be associated with increased EC risk (Lindemann* et al*. 2009, Gong *et al*. 2016). Importantly, concentrations of the cholesterol metabolite 27HC are increased in postmenopausal women (Burkard* et al*. 2007) and are associated with increased risk of breast cancer. Several studies have identified that 27HC has an adverse impact on breast cancer (Nelson *et al*. 2013, Wu *et al*. 2013) but whether 27HC can affect EC has not been investigated previously.

In light of these studies, we hypothesised that 27HC signalling could contribute to the aetiology of endometrial cancer and influence disease progression, and we investigated this using both archival human tissue as well as cell lines that are derived from different grades of EC. We obtained new evidence for expression of the enzymes required for the both the synthesis (CYP27A1) and breakdown (CYP7B1) of 27HC. As concentrations of *CYP7B1* mRNAs were significantly decreased in poorly compared to moderately differentiated cancers and expression of *CYP27A1* did not change significantly across EC grades; we believe this would favour increased bioavailability of 27HC with increasing grade. These findings appear to parallel those reported for ER+ breast cancer where decreased expression of CYP7B1 and increased CYP27A1 has been reported in tumours compared to normal breast tissues (Wu *et al*. 2013). Furthermore, we found that the endogenous receptor for 27HC, LXR, was immunolocalised to stage 1 cancers and was expressed throughout the tissue and localised to the nuclei of both stromal and epithelial cells.

We sought to establish if 27HC could alter responses in EC cells by acting via its cognate receptor, LXR, or via estrogen receptors, which are known to regulate endometrial proliferation. 27HC activated LXR-dependent transcription in all cell lines tested. In contrast, we found that 27HC activated ERE-dependent reporter gene expression in well-differentiated cancer cells (Ishikawa; ERα+ERβ+) but not in those from moderately (RL95; ERα^low^ERβ+) or poorly differentiated cancers (MFE280; ERα^low^ERβ^low^). However, 27HC increased proliferation of both Ishikawa and RL95 cells but not MFE280 cells consistent with reported ER expression in these cell lines (Johnson *et al*. 2007, Yang *et al*. 2008, Li *et al*. 2014). Our immunohistochemistry analysis (Supplementary Fig. 2) supported these *in vitro* findings. We found that the proliferation marker Ki67 co-localised with ERα in well- and moderately differentiated cancers consistent with a key role for this receptor in mediating endometrial epithelial cell proliferation (Lubahn *et al*. 1993, Frasor *et al*. 2003). In poorly differentiated cancers, ERα was not detected consistent with previous reports (Collins *et al*. 2009). It has been reported that 27HC, acting as a SERM, can impact on ERα- or ERβ1-dependent regulation of cell function (He & Nelson 2017) and the oestrogenic effects of 27HC could therefore be mediated via either ER isoform in EC cells. In endometrial endothelial cells, which express ERβ but not ERα, oestrogenic effects are mediated via ERβ tethered to Sp1 and not via direct binding to ERE (Greaves* et al*. 2013). Furthermore, it has been reported that 27HC promotes proliferation of ERα-positive LNCaP prostate cancer cells via ERβ (Lau* et al*. 2000, Raza* et al*. 2017) which may account for the apparent discrepancy between ERE reporter assay and cell proliferation responses in RL95 cells observed in the current study. Taken together, these findings reveal the potential for 27HC generated within the EC tissue microenvironment to influence ER-dependent transcription and proliferation via ERs expressed in early-grade stage 1 EC.

Although the association between ERs and endometrial proliferation is well recognised, there is limited data investigating the role of LXR in this process. Expression of LXRα and LXRβ mRNA has been previously reported in endometrium and myometrium of mice (Mouzat* et al*. 2007) and 27HC is reported to increase mouse uterine weight, consistent with an uterotrophic action; however, whether this was mediated via ER or LXR was not investigated (Wu *et al*. 2013). In mice, targeted ablation of the receptor subtypes revealed that *Lxrα−/−* but not *Lxrβ−/−* females had reduced endometrial areas compared to wildtype mice consistent with a role for LXRα in promoting endometrial growth/proliferation in that species (Mouzat *et al*. 2007). In the current study, we found that LXR co-localised with the proliferation marker Ki67 in well-differentiated but not moderate- or poorly differentiated EC tissues. *In vitro* assays verified this finding as the synthetic LXR agonist GW3965 had a cell-selective impact on the EC cell lines. In Ishikawa cells GW3965 increased proliferation, whereas in RL95 and MFE280 cells equimolar concentrations of agonist blocked proliferation. Given that LXR expression was detected in all grades of EC, this may suggest LXR could be an effective therapeutic target in some ECs, albeit in a grade-dependent context. Indeed, GW3965 is reported to abrogate E2-mediated increases in MCF7 breast cancer cell proliferation and has been proposed as an anti-proliferative ligand in this context (Vedin *et al*. 2009).

LXR classically acts as a heterodimeric partner of retinoid X receptor (RXR). RXR is expressed in the nuclei of endometrial epithelial cells throughout the menstrual cycle (Fukunaka* et al*. 2001) as well as in EC tissues (Nickkho-Amiry* et al*. 2012). Interestingly, LXR-RXR functions as a ‘permissive’ heterodimer and binding of either an LXR agonist or the RXR agonist 9-*cis* retinoic acid activates transcription, whilst agonism of both dimer partners has an additive effect on activation. Assessment of RXR isoforms in the cell lines used in the current study demonstrated differential expression of RXRs in Ishikawa, RL95 and MFE280 cells, which may account for the distinct responses of these cell lines in response to GW3965 treatment (Supplementary Fig. 5). *NR2B1* (RXRα) mRNA expression was greatest in RL95 cells whilst *NR2B2* (RXRβ) was detected in all cell lines. Notably, mRNA expression of *NR2B3* (RXRγ) was not detected in RL95 cells but was abundant in MFE280 cells. Whether changes in the constitution of the receptor isoforms that contribute to the LXR:RXR heterodimer affect responses requires further investigation; however, previous studies demonstrate that targeting retinoid signalling may affect proliferation of EC cells. Notably, retinoic acid (RA) signalling via retinoic acid receptor (RAR) and RXR is reported to inhibit Ishikawa cell proliferation by inducing cell cycle arrest (Cheng* et al*. 2011) and fenretinide, a synthetic derivative of RA, induced apoptosis of Ishikawa cells (Mittal* et al*. 2014). These results suggest targeting LXR-dependent signalling with LXR and/or RXR agonists could inhibit proliferation in EC and cancer progression.

Changes in the local inflammatory environment that occur during development and progression of EC may also increase exposure to 27HC due to infiltration of inflammatory cells. We have previously demonstrated that infiltration of immune cells is increased in EC tissues compared to controls. Notably, the numbers of macrophages, neutrophils and dendritic cells were significantly increased in EC tissues (Wallace* et al*. 2010) consistent with 27HC-dependent increases in migration of bone marrow-derived CD11b^+^ cells reported in *in vitro* assays (Raccosta* et al*. 2013). In addition, 27HC increases secretion of CCL2 from macrophages which enhances recruitment of monocytes (Kim* et al*. 2013) and can also upregulate ER-dependent expression of pro-inflammatory genes (Umetani *et al*. 2014). Notably, as Cyp27a1 is reported to be abundant in macrophages (2), these cells may also contribute to an increase in 27HC within the tumour microenvironment. In support of this idea, increased 27HC concentrations have been reported in breast cancer tumours (Wu *et al*. 2013) and increased concentrations of cholesterol have been reported in tumours of various cancer types although they have not been directly measured in EC. 27HC can also promote secretion of TNFA and IL6 from macrophages and TNFA is reported to increase proliferation of human endometrial glandular epithelial cells (Nair* et al*. 2013). Thus, although in the current study we only investigated the direct impact of 27HC on proliferation of EC epithelial cells, 27HC may also exacerbate changes within the tissue microenvironment by modulating inflammatory responses, and this merits further investigation in animal models.

## Summary

In the current study, we provide the first evidence to support a mechanistic link between exposure to elevated cholesterol, biosynthesis of 27HC and EC. Analysis of human stage 1 endometrial adenocarcinomas revealed expression of the key metabolising enzymes of 27HC was altered in EC consistent with increased exposure to 27HC as EC progresses from well to poorly differentiated. Although survival rates for EC are high, incidence rates are increasing in line with rates of obesity and a rising incidence in pre- and peri-menopausal women creates unique therapeutic challenges. Based on our novel findings, we propose that exposure to 27HC may influence disease development/progression by activating ER-dependent pathways to increase epithelial cell proliferation. These results suggest strategies that seek to limit exposure to 27HC through lifestyle modification, lipid-lowering drugs such as statins or novel therapeutics that target 27HC synthesis (CYP27A1 inhibitors) may be effective in reducing endometrial proliferation in women at increased risk of developing EC. Taken together, our novel findings suggest that altered cholesterol metabolism, and aberrant exposure to 27HC, may contribute to the development and/or progression of EC.

## Supplementary Material

Supporting Figure 1

Supporting Figure 2

Supporting Figure 3

Supporting Figure 4

Supporting Figure 5

Supporting Information

Supporting Table 1

Supporting Table 2

Supporting Table 3

Supporting Table 4

## Declaration of interest

The authors declare that there is no conflict of interest that could be perceived as prejudicing the impartiality of the research reported.

## Funding

Studies undertaken in the Saunders laboratory were supported by MRC Programme Grant G1100356/1 (P T K S) and CRUK development fund (D A G and P T K S).

## Author contribution statement

Experimental design; D A G, F C and P T K S, experimental procedures; D A G, F C, A E Z and F L C, manuscript preparation; D A G, F C and P T K S.
