# Supplementary figures and images for: The impact of 27-hydroxycholesterol on endometrial cancer proliferation
Source: Endocr Relat Cancer. 2018 Jan 25;25(4):381–91. doi: 10.1530/ERC-17-0449 (PMC5847183; doi:10.1530/ERC-17-0449)

**Well**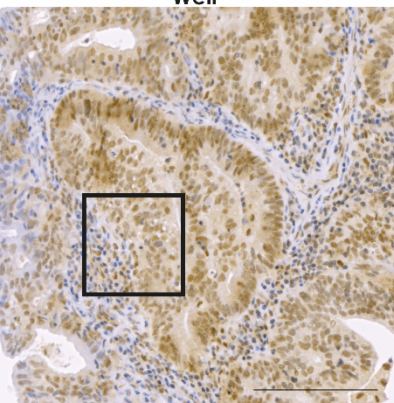**Mod**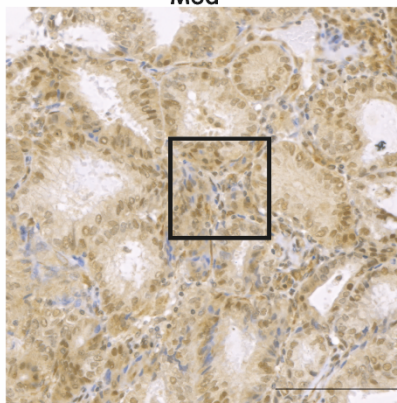**Poor**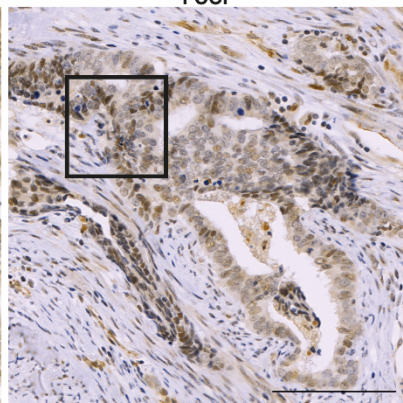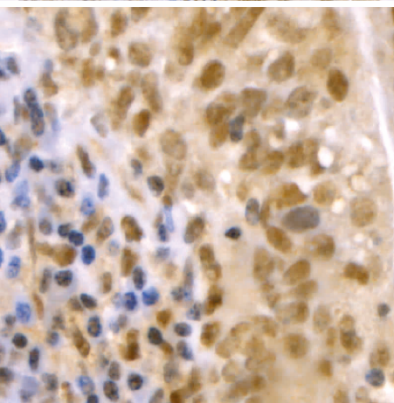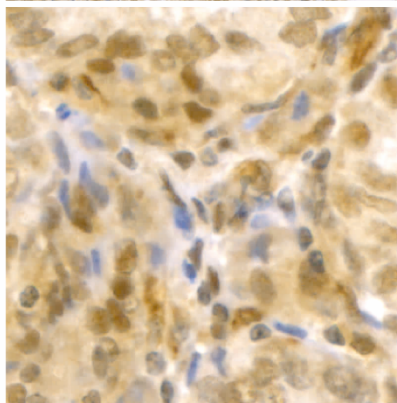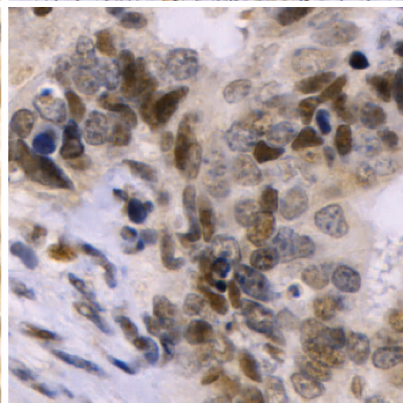

Supplement: Supporting Figure 1 [file erc-25-381-s001.pdf]

Well

Moderate

Poor

1614

931

910

871

739

2178

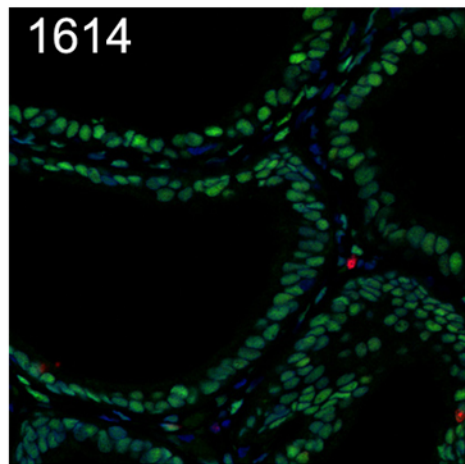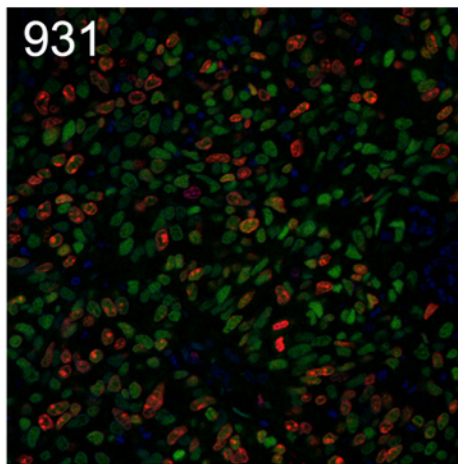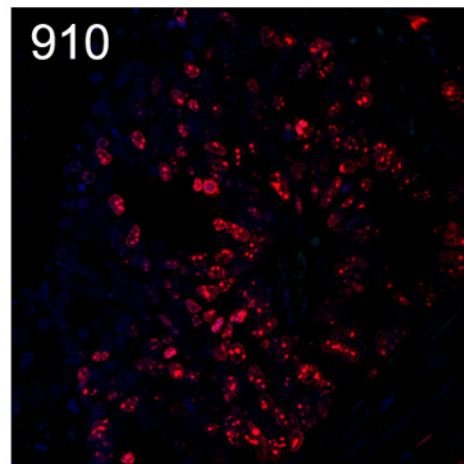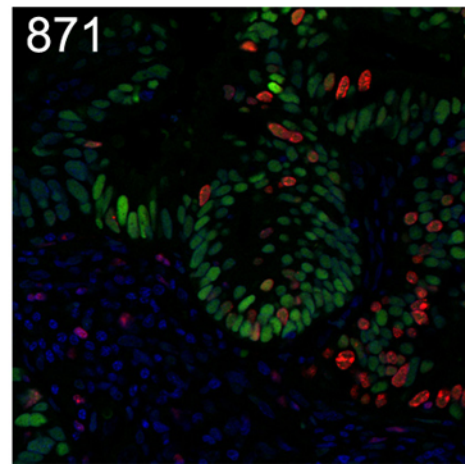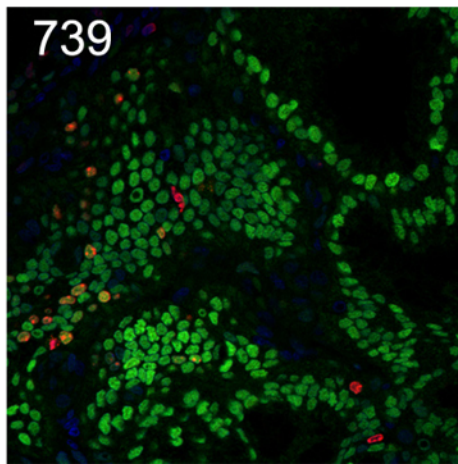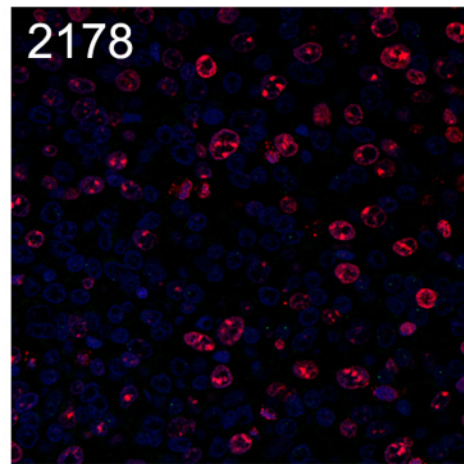

Supplement: Supporting Figure 2 [file erc-25-381-s002.pdf]

**A**

LXRA (50kDa)

ACTIN (43kDa)

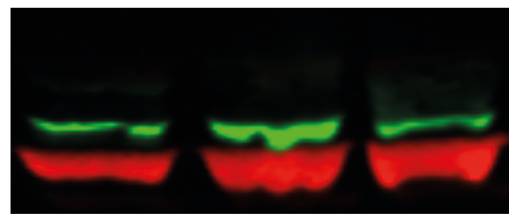

ISH

RL95

MFE

**B**

LXRB (56kDa)

ACTIN (43kDa)

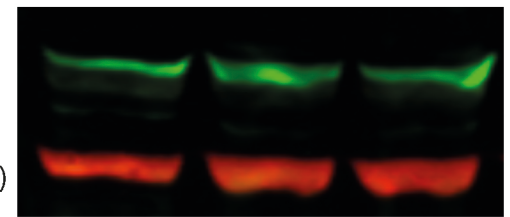

ISH

RL95

MFE

**C**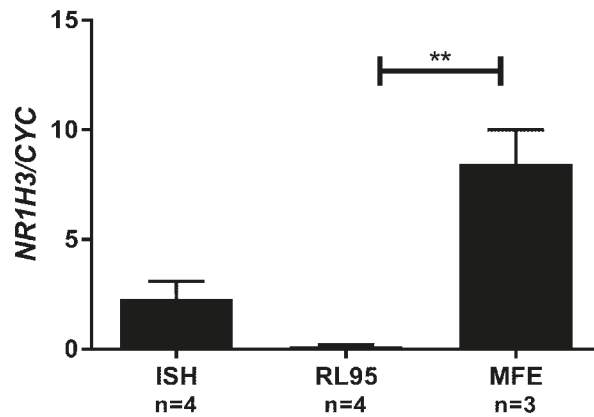**D**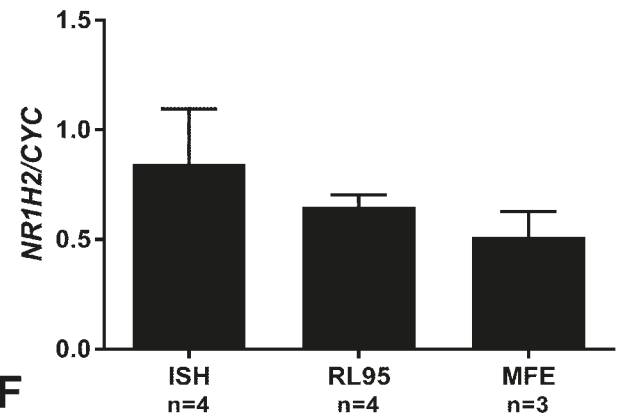**E**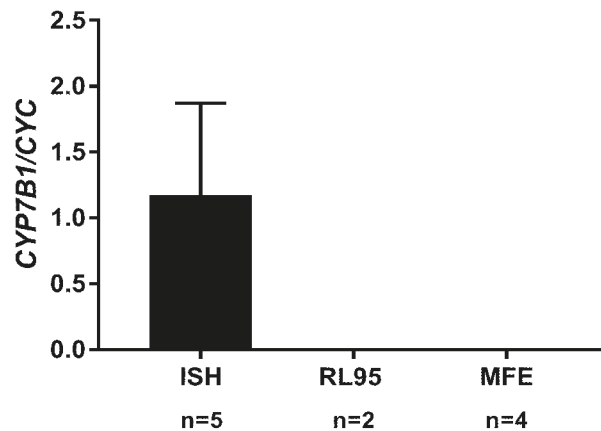**F**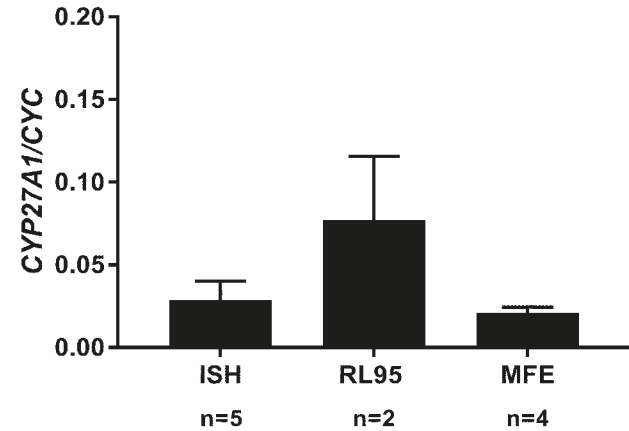

Supplement: Supporting Figure 3 [file erc-25-381-s003.pdf]

**A**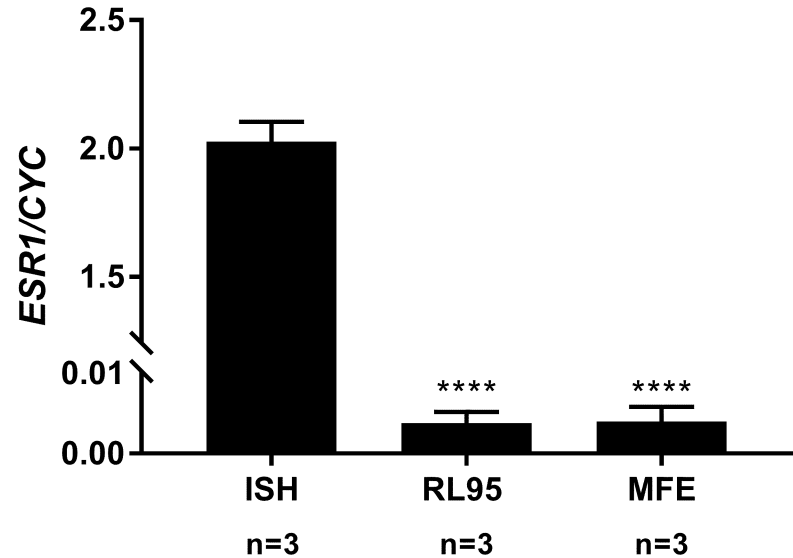**B**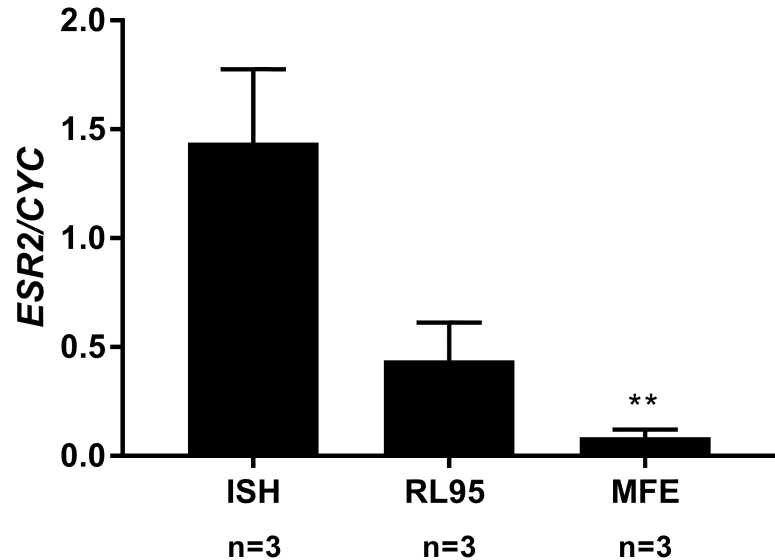

Supplement: Supporting Figure 4 [file erc-25-381-s004.pdf]

**A**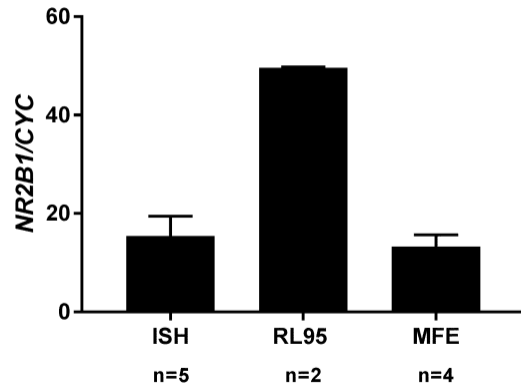**B**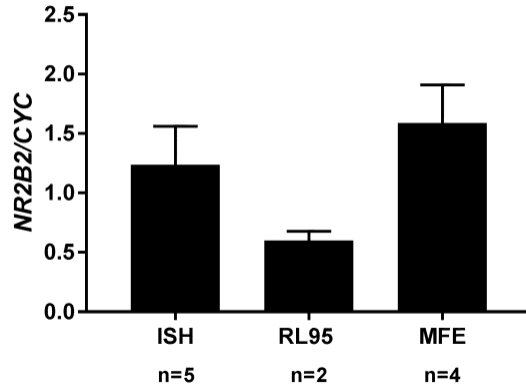**C**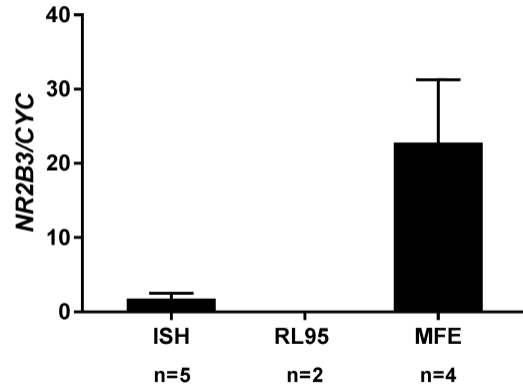

Supplement: Supporting Figure 5 [file erc-25-381-s005.pdf]
