# Supplementary material for: The impact of 27-hydroxycholesterol on endometrial cancer proliferation
Source: Endocr Relat Cancer. 2018 Jan 25;25(4):381–91. doi: 10.1530/ERC-17-0449 (PMC5847183; doi:10.1530/ERC-17-0449)
Supplement: Supporting Information [file erc-25-381-s006.pdf]

Supplementary Figure 1. Expression of LXR in endometrial cancer. The expression of LXR (antibody identified both isoforms) was assessed by immunohistochemistry in endometrial cancer tissue sections from well, moderate and poorly differentiated cancers. LXR was expressed throughout the tissue and localised to the nuclei of both stromal and epithelial cells (brown) in all cancer grades. Although staining was most intense in the nuclei, cytoplasmic staining was also detected possibly as a result of altered nuclear-cytoplasmic shuttling in cancer tissues. Cytoplasmic staining was not detected using immunofluorescence (Figure 2). Images representative of at least 3 different patients per cancer grade. Nuclear counterstain Haematoxylin (blue), scale bar 100µm.

Supplementary Figure 2. Expression of ERα and the proliferation marker Ki67 in endometrial cancer. The expression of ERα and the proliferation marker Ki67 was assessed by immunohistochemistry in endometrial cancer tissue sections. In well- and moderately-differentiated cancers (**1614, 871; 931, 739**), ERα was expressed throughout the tissue and localised to the nuclei of both stromal and epithelial cells (green staining). Nuclear immunoexpression of Ki67 (red staining) was detected and co-localised with ERα expression although some Ki67-positive cells were ERα-negative. In poorly differentiated cancers (**910, 2178**), ERα was not detected but Ki67 was abundantly expressed. Images representative of at least 3 different patients per cancer grade, samples collected and processed as described in *Collins et al. BMC Cancer* 2009, **9**:330. Nuclear counterstain DAPI (blue).

Supplementary Figure 3. Endometrial epithelial cancer cells lines express both isoforms of LXR and enzymes required for regulation of 27HC. Expression of LXRα (**A**) and LXRβ (**B**) protein was assessed by Western blot; mRNA expression of LXR isoforms; **C** *NR1H3* (LXRα) and **D** *NR1H2* (LXRβ) and the enzymes; **E** *CYP27A1* and **F** *CYP7B1* relative to internal control *CYC* was assessed by qPCR in endometrial cancer cell lines; Ishikawa (ISH; n=4-5)), RL95 (n=2-4) and MFE280 (n=3-4), representative of well-, moderately- and poorly-differentiated cancer respectively. Western blotting was performed using 20µg/lane total cell lysates; membranes were probed with mouse anti-LXRα (Green, Predicted molecular weight: 50 kDa; abcam ab41902), mouse anti-LXRβ (Green, Predicted molecular weight: 50 kDa; Invitrogen 418400), loading control was goat anti-actin (Santa-

Cruz biotech sc-1616; predicted molecular weight 43kDa; red). Membranes were incubated with species-specific fluorescent-conjugated secondary antibodies and visualised using the Licor Odyssey system (Licor). **p<0.01. Kruskal-Wallis test with multiple comparisons. Statistically significant comparison RL95 vs MFE280. All data are presented as mean ± SEM.

Supplementary Figure 4. Endometrial epithelial cancer cells lines express both ER isoforms. Expression mRNAs encoding ER isoforms; **A** *ESR1* (ERα) and **B** *ESR2* (ERβ) relative to internal control *CYC* was assessed by qPCR in endometrial cancer cell lines; Ishikawa (ISH; n=3), RL95 (n=3) and MFE280 (n=3), representative of well-, moderately- and poorly-differentiated cancer respectively. **p<0.01, **** p<0.0001. Kruskal-Wallis test with multiple comparisons. Statistical comparisons relative to Ishikawa (ISH). All data are presented as mean ± SEM.

Supplementary Figure 5. Endometrial epithelial cancer cells lines differentially express RXR isoforms. mRNA expression of RXR isoforms; **A** *NR2B1* (RXRα) and **B** *NR2B2* (RXRβ) and **C** *NR2B3* (RXRγ) relative to internal control gene *CYC* was assessed by qPCR in endometrial cancer cell lines; Ishikawa (ISH; n=6), RL95 (n=2) and MFE280 (n=4), representative of well-, moderately- and poorly-differentiated cancer respectively. All data are presented as mean ± SEM.
