# Supplementary material for: The impact of 27-hydroxycholesterol on endometrial cancer proliferation
Source: Endocr Relat Cancer. 2018 Jan 25;25(4):381–91. doi: 10.1530/ERC-17-0449 (PMC5847183; doi:10.1530/ERC-17-0449)
Supplement: Supporting Table 1 [file erc-25-381-t001.pdf]

Supplementary Table 1 - Patient characteristics

	Stage 1 Endometrial carcinoma grades			
	**Well**	**Moderate**	**Poor**	**P value**
**Average age (+/- SEM)**	63.6 +/- 2.13	63.5 +/- 1.95	69.4 +/- 2.14	0.0706
**Average BMI (+/- SEM)**	31.3 +/- 2.58	32.6 +/- 2.79	36.2 +/- 3.99	0.6442

LMP –last menstrual period, statistical comparison using Kruskal-Wallis test.
