# Supplementary material for: The impact of 27-hydroxycholesterol on endometrial cancer proliferation
Source: Endocr Relat Cancer. 2018 Jan 25;25(4):381–91. doi: 10.1530/ERC-17-0449 (PMC5847183; doi:10.1530/ERC-17-0449)
Supplement: Supporting Table 2 [file erc-25-381-t002.pdf]

Supplementary Table 2 – Sample numbers for qPCR analysis

**Gene Symbol**	**Well differentiated (G1)**	**Moderately differentiated (G2)**	**Poorly differentiated (G3)**
NR1H3	12	44	26
NR1H2	30	64	24
CYP7B1	19	54	32
CYP27A1	19	54	32
