# Supplementary material for: The impact of 27-hydroxycholesterol on endometrial cancer proliferation
Source: Endocr Relat Cancer. 2018 Jan 25;25(4):381–91. doi: 10.1530/ERC-17-0449 (PMC5847183; doi:10.1530/ERC-17-0449)
Supplement: Supporting Table 3 [file erc-25-381-t003.pdf]

Supplementary Table 3 - Primers and probes for qRTPCR.

**Gene Symbol**	**5' to 3'**	**3' to 5'**	**UPL probe**
NR1H3	*catcctcttctcccagcaag*	*cattaccaaggcactgtcca*	49
NR1H2	*ttcacctacagcaaggacga*	*gaactcgaagatggggttga*	39
CYP7B1	*ccccttaaggttcatgaaaaca*	*tggaaggggtccaggataa*	79
CYP27A1	*ggctggagtggacacgac*	*accacacccaccacttcct*	31
ESR1	*aaccagtgcaccattgataaaa*	*tcctcttcggtcttttcgtatc*	69
ESR2	*gctcctgtcccacgtcag*	*tgggcattcagcatctcc*	62
NR2B1	*acatgcagatggacaagacg*	*tcgagagccccttggagt*	26
NR2B2	*agctcccccaggattctc*	*ccagggagtgacactgttgag*	66
NR2B3	*cctgcgagccattgtactct*	*aagggtggcataaaccttctc*	39
