# Supplementary material for: The impact of 27-hydroxycholesterol on endometrial cancer proliferation
Source: Endocr Relat Cancer. 2018 Jan 25;25(4):381–91. doi: 10.1530/ERC-17-0449 (PMC5847183; doi:10.1530/ERC-17-0449)
Supplement: Supporting Table 4 [file erc-25-381-t004.pdf]

Supplementary Table 4 – antibodies used in Immunohistochemistry and Western blot (WB) analysis

**Antibody name**	**Species raised**	**Supplier**	**Cat No**	**Dilution**
LXRα/β Antibody (G-10)	Mouse monoclonal	Santa Cruz bt	sc-271064	1:530 (DAB)<br>1:1400
Estrogen receptor alpha	Mouse monoclonal	Vector	VP-614	1:20
Ki67	Rabbit polyclonal	Abcam	Ab15580	1:2000
LXRα (WB)	Mouse monoclonal	Abcam	ab41902	1:1000
LXRβ (WB)	Mouse monoclonal	Invitrogen	418400	1:500
ACTIN (I-19) (WB)	Goat polyclonal	Santa Cruz bt	sc-1616	1:500
